# Complete Genome Sequences of Two Klebsiella pneumoniae Phages Isolated as Part of an International Effort

**DOI:** 10.1128/MRA.00843-19

**Published:** 2019-09-19

**Authors:** Ortal Yerushalmy, Shunit Coppenhagen-Glazer, Ran Nir-Paz, Henni Tuomala, Mikael Skurnik, Saija Kiljunen, Ronen Hazan

**Affiliations:** a Institute of Dental Sciences, The Hebrew University of Jerusalem, Jerusalem, Israel; b Department of Clinical Microbiology and Infectious Diseases, Hadassah-Hebrew University Medical Center, Jerusalem, Israel; c Division of Clinical Microbiology, Helsinki University Hospital, HUSLAB, Helsinki, Finland; d Department of Bacteriology and Immunology, Medicum, Human Microbiome Research Program, Faculty of Medicine, University of Helsinki, Helsinki, Finland; Queens College

## Abstract

We report the genomic sequences of phages KpCHEMY26 and KpGranit, isolated in Israel during a worldwide effort against a multidrug- and phage-resistant strain of Klebsiella pneumoniae from a patient in Finland. These results demonstrate the importance of an efficient worldwide network for collaborating in personalized therapy for infectious diseases.

## ANNOUNCEMENT

Klebsiella pneumoniae, a Gram-negative bacterium, is one of the Enterococcus faecium, Staphylococcus aureus, Klebsiella pneumoniae, Acinetobacter baumannii, Pseudomonas aeruginosa, and *Enterobacter* (ESKAPE) species’ most problematic multidrug-resistant pathogens (1, 2). It is associated with invasive infections, including bacteremia, sepsis, and abdominal and respiratory system infections (3), and is thus a major target for phage therapy (4).

In 2018, authors from the University of Helsinki received a K. pneumoniae patient isolate that was resistant to all the antibiotic agents and phages tested. They approached Phage Directory, a worldwide network of phage labs (https://phage.directory/), with a request for phages against this isolate. Authors from The Hebrew University of Jerusalem collected environmental samples from various sources in Israel and incubated them with the target bacterium in LB broth for 24 hours. The samples were centrifuged (7,800 × *g* for 10 min) and passed through filters (0.22 μm), and a plaque assay was performed as described (5). One of the soil samples collected near Jerusalem yielded two phages, designated KpCHEMY26 and KpGranit.

For DNA isolation, both phages were produced by adding the phage to mid-log cultures of K. pneumoniae at a multiplicity of infection (MOI) of 1. The cultures were incubated for 24 h and yielded titers of 10^9^ PFU/ml after filtering (0.22-μm filter).

The DNA of KpCHEMY26 was purified using the phage DNA isolation kit (Norgen Biotek), and libraries were prepared with a Nextera XT DNA kit (catalog number FC-131-1096; Illumina, San Diego, CA). Normalization, pooling, and tagging were performed in a common flow cell with 1 × 150-bp paired-end reads, which were used for sequencing with the Illumina NextSeq 500 platform (6, 7). Sequencing was performed in the sequencing unit of The Hebrew University of Jerusalem at the Hadassah Campus.

The DNA of phage KpGranit was isolated using phenol-chloroform extraction and ethanol precipitation (8), polished with Vivacon 500 100-kDa ultrafiltration columns (Sartorius), and sequenced by Eurofins GATC Biotech. The quality of the reads was assessed using FastQC (https://www.bioinformatics.babraham.ac.uk/projects/fastqc/). The trimming and *de novo* assembly were performed using Geneious Prime 2019 and its plugins. For assembly, the Geneious assembler and the SPAdes plugin of Geneious were used with default parameters (medium-low sensitivity).

Annotation was performed using RAST (9), PHASTER (https://phaster.ca) (9), and BLAST (10) servers. tRNA genes were predicted using tRNAscan-SE version 1.21 (11) and ARAGORN (12). Terminal repeats were predicted using PhageTerm (13).

Phage KpCHEMY26 contains a linear genome of 70,678 bp, which was assembled to a single contig from 1,608,406 of 5,396,479 reads with a mean coverage of 2,473.1× (±685.5). It has 81 putative open reading frames and 11 tRNA genes. Among the identified genes are replication, structural, and lysis genes. KpCHEMY26 has an identity of 95.01% for K. pneumoniae phage Pylas (GenBank accession number MH899585).

Phage KpGranit contains a linear genome of 122,710 bp assembled to a single contig from 1,097,946 of 1,118,794 reads with a mean coverage of 1,474.6× (±506.9). Its genome has direct terminal repeats, 160 putative open reading frames, and 26 tRNA genes. Most of these genes are hypothetical, and among the identified genes are replication, structural, and lysis genes. KpGranit displays genome-wide similarity to the following T5-like phages, mainly of K. pneumoniae: vB_Kpn_IME260 (96.52% identity; GenBank accession number NC_041899), Sugarland (96.51% identity; NC_042093), vB_KpnS_FZ41 (94.39% identity; MK521907), and Spivey (92.32% identity; MK630230). The two phages belong to the *Caudovirales* order; KpCHEMY26 is related to the *Podoviridae* family, and KpGranit is related to the *Siphoviridae* family. Interestingly, both have a significantly lower GC content (KpGranit, 45%; KpCHEMY26, 42%) than the K. pneumoniae host (∼57.6%), perhaps indicating that they originated from another bacterium. Genes involved in lysis (14), including holin, endolysin, cell wall hydrolase, and O- and I-spanins, were identified in both phages.

In both phages, no known virulence or lysogeny genes were detected using the Virulence Factors Database (15). These results demonstrate that with an international collaborative effort, lytic phages can be found against even highly resistant bacteria (15).

### Data availability.

The genome sequences of *Klebsiella* phages KpGranit and KpCHEMY26 have been deposited in NCBI GenBank under the accession numbers MN163280 and MN163281, respectively. The raw data were deposited in the NIH BioSample database project PRJNA555313 with the accession numbers SAMN12307310 for KpCHEMY26 and SAMN12307311 for KpGranit.

## References

[1] Gomez-Simmonds A, Uhlemann AC 2017 Clinical implications of genomic adaptation and evolution of carbapenem-resistant *Klebsiella pneumoniae*. J Infect Dis 215:S18–S27. doi:10.1093/infdis/jiw378. 28375514 PMC5853309

[2] Pendleton JN, Gorman SP, Gilmore BF 2013 Clinical relevance of the ESKAPE pathogens. Expert Rev Anti Infect Ther 11:297–308. doi:10.1586/eri.13.12. 23458769

[3] Struve C, Krogfelt KA 2004 Pathogenic potential of environmental *Klebsiella pneumoniae* isolates. Environ Microbiol 6:584–590. doi:10.1111/j.1462-2920.2004.00590.x. 15142246

[4] Mulani MS, Kamble EE, Kumkar SN, Tawre MS, Pardesi KR 2019 Emerging strategies to combat ESKAPE pathogens in the era of antimicrobial resistance: a review. Front Microbiol 10:539. doi:10.3389/fmicb.2019.00539. 30988669 PMC6452778

[5] Khalifa L, Gelman D, Shlezinger M, Dessal AL, Coppenhagen-Glazer S, Beyth N, Hazan R 2018 Defeating antibiotic- and phage-resistant *Enterococcus faecalis* using a phage cocktail *in vitro* and in a clot model. Front Microbiol 9:326. doi:10.3389/fmicb.2018.00326. 29541067 PMC5835721

[6] Khalifa L, Coppenhagen-Glazer S, Shlezinger M, Kott-Gutkowski M, Adini O, Beyth N, Hazan R 2015 Complete genome sequence of *Enterococcus* bacteriophage EFLK1. Genome Announc 3:e01308-15. doi:10.1128/genomeA.01308-15. 26586876 PMC4653778

[7] Alkalay S, Sternberg S, Coppenhagen-Glazer S, Hazan R 2018 Complete genome sequences of three *Bacillus anthracis* bacteriophages. Genome Announc 6:e01164-17. doi:10.1128/genomeA.01164-17. 29301897 PMC5754477

[8] Sambrook J, Fritsch E, Maniatis T 1989 Chapter 3, Working with bacteriophage M13. Section 3, Preparation of double-stranded (replicative form) bacteriophage M13 DNA, p 3.23–3.26. *In* Molecular cloning: a laboratory manual. Cold Spring Harbor Laboratory Press, New York, NY.

[9] Brettin T, Davis JJ, Disz T, Edwards RA, Gerdes S, Olsen GJ, Olson R, Overbeek R, Parrello B, Pusch GD, Shukla M, Thomason JA, Stevens R, Vonstein V, Wattam AR, Xia F 2015 RASTtk: a modular and extensible implementation of the RAST algorithm for building custom annotation pipelines and annotating batches of genomes. Sci Rep 5:8365. doi:10.1038/srep08365. 25666585 PMC4322359

[10] Johnson M, Zaretskaya I, Raytselis Y, Merezhuk Y, McGinnis S, Madden TL 2008 NCBI BLAST: a better Web interface. Nucleic Acids Res 36:W5–W9. doi:10.1093/nar/gkn201. 18440982 PMC2447716

[11] Lowe TM, Chan PP 2016 tRNAscan-SE On-line: integrating search and context for analysis of transfer RNA genes. Nucleic Acids Res 44:W54–W57. doi:10.1093/nar/gkw413. 27174935 PMC4987944

[12] Laslett D, Canback B 2004 ARAGORN, a program to detect tRNA genes and tmRNA genes in nucleotide sequences. Nucleic Acids Res 32:11–16. doi:10.1093/nar/gkh152. 14704338 PMC373265

[13] Garneau JR, Depardieu F, Fortier L-C, Bikard D, Monot M 2017 PhageTerm: a tool for fast and accurate determination of phage termini and packaging mechanism using next-generation sequencing data. Sci Rep 7:8292. doi:10.1038/s41598-017-07910-5. 28811656 PMC5557969

[14] Cahill J, Young R 2019 Phage lysis: multiple genes for multiple barriers. Adv Virus Res 103:33–70. doi:10.1016/bs.aivir.2018.09.003. 30635077 PMC6733033

[15] Chen L, Xiong Z, Sun L, Yang J, Jin Q 2012 VFDB 2012 update: toward the genetic diversity and molecular evolution of bacterial virulence factors. Nucleic Acids Res 40:D641–D645. doi:10.1093/nar/gkr989. 22067448 PMC3245122

